# Celastrol ameliorates inflammation through inhibition of NLRP3 inflammasome activation

**DOI:** 10.18632/oncotarget.18619

**Published:** 2017-06-27

**Authors:** Xianjun Yu, Qun Zhao, Xixi Zhang, Haiwei Zhang, Yongbo Liu, Xiaoxia Wu, Ming Li, Xiaoming Li, Jingxuan Zhang, Xuzhi Ruan, Haibing Zhang

**Affiliations:** ^1^ Key Laboratory of Nutrition and Metabolism, Institute for Nutritional Sciences, Shanghai Institutes for Biological Sciences, Chinese Academy of Sciences, University of Chinese Academy of Sciences, Shanghai 200031, China; ^2^ Laboratory of Inflammation and Molecular Pharmacology, School of Basic Medical Sciences, Hubei University of Medicine, Shiyan 442000, China

**Keywords:** celastrol, NLRP3, inflammasome, inflammatory disease

## Abstract

Celastrol exhibits potential anti-inflammatory activity in a variety of inflammatory diseases, but the mechanism remains poorly understood. Activation of NLRP3 inflammasome is involved in multiple inflammatory diseases. Here, we show that celastrol abolishes the NLRP3 inflammasome activation, inhibits subsequent caspase-1 activation and IL-1β secretion both *in vitro* and *in vivo*. Notably, interruption of ASC oligomerization and autophagy activation are involved in NLRP3 inflammasome inactivation by celastrol. Importantly, *in vivo* results indicate that celastrol attenuates NLRP3 inflammasome-dependent inflammation diseases via autophagy-related pathway. Our results thus reveal celastrol as an inhibitor of NLRP3 inflammasome, implying the potential for clinical use of celastrol in treatment of NLRP3 inflammasome-driven inflammatory diseases.

## INTRODUCTION

Celastrol, a natural triterpene, is an active ingredient isolated from the root bark of the traditional Chinese medicinal plant *Tripterygium wilfordii* Hook F (thunder god vine) [1]. Celastrol has potent antitumor activities, which include proliferation inhibition, apoptosis induction, invasion and angiogenesis suppression [2–7]. Recently, Liu *et al*. have reported that celastrol ameliorates obesity via increasing leptin sensitivity [8]. In addition, celastrol exhibits potent anti-inflammatory activities in various experimental models [9]. Celastrol inhibits cytokine expression by interrupting NF-κB signaling pathway [10]. Celastrol also exhibits multiple beneficial effects on autoimmune diseases, including acute and chronic inflammation, neurodegenerative diseases [11–13]. Although the use of celastrol for treatment of inflammatory disorders is promising, the anti-inflammatory mechanism of celastrol are not fully understood.

The NLRP3 inflammasome is a multiprotein complex composed of NLRP3, ASC and Caspase-1 [14]. It is a central innate immune sensor that is triggered by pathogen infection or endogenour “danger” signal, such as infection, tissue damage and metabolic dysregulation [15]. The activation of NLRP3 inflammasome promotes the activation of caspase-1, the maturation and release of several pro-inflammatory cytokines, including IL-1β and IL-18 [16]. Enhanced activity of NLRP3 inflammasome activity is associated with several inflammatory diseases, including endotoxin shock [17], ulcerative colitis [18], Alzheimer disease [19], obesity [20], type 2 diabetes [21], atherosclerosis [22] and gout [23]. Thus, the activation of NLRP3 inflammasome has been controlled tightly *in vivo*, several endogenous regulators and small molecules have been shown to suppress NLRP3 inflammasome. Nitric oxide exhibits inhibitory effect on NLRP3 inflammasome [17], while the ketone bodies b-hydroxybutyrate and dopamine have been identified as endogenous negative regulator of NLRP3 inflammasome activation [24–25]. Moreover, exogenous omega-3 fatty acids and MCC950 have been reported to inhibit the activity of NLRP3 inflammasome [26–27]. Thus, characterization of the NLRP3 inflammasome inhibitor may provide novel insights into the control of inflammatory processes [28].

Celastrol has been reported to exert anti-inflammatory effects by inhibiting the production of proinflammatory cytokines, including IL-1β [11], and this prompted us to investigate whether the effects of celastrol are mediated through inhibition NLRP3 inflammasome activation. In this study, we demonstrated that celastrol is a potent inhibitor of the NLRP3 inflammasome. Furthermore, we found celastrol prohibited LPS-induced systemic inflammation and DSS-induced colitis via the inhibition of NLRP3 inflammasome activation *in vivo*. Our findings pave a novel avenue for the anti-inflammatory effect of celastrol.

## RESULTS

### Celastrol suppresses IL-1β secretion and caspase-1 activation in mouse macrophages

**Figure 1:**
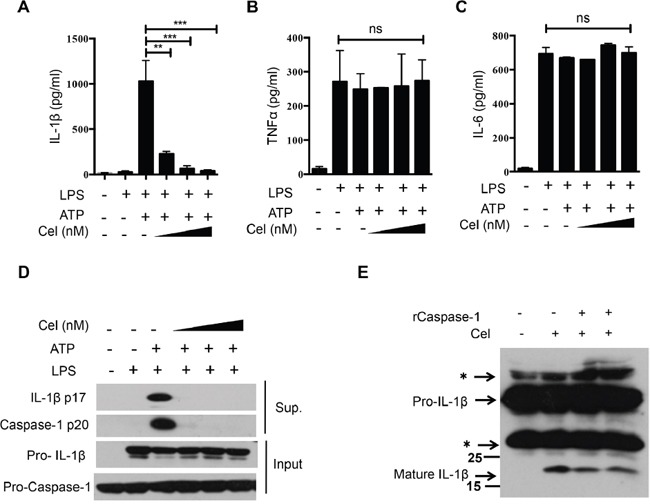
Celastrol suppresses IL-1β secretion and caspase-1 activation in mouse macrophages **(A-C)** LPS-primed peritoneal macrophages were treated with various doses of celastrol (125, 250, 500 nM) for 30 min, following by treatment with ATP for 30 min. Supernatants were analyzed by ELISA for IL-1β, TNF-α and IL-6 release. **(D)** LPS-primed peritoneal macrophages were treated with various doses of celastrol (125, 250, 500 nM,) for 30 min, following by treatment with ATP for 30 min. Supernatants (SN) and cell extracts (Input) were analyzed by immunoblotting. **(E)** Peritoneal macrophages were primed with LPS for 3 h, following by treatment with celastrol (250 nM) for another 30 min. The sucrose lysates were incubated with recombinant active caspase-1 for 3 h. Immunoblotting analysis of IL-1β. The asterisk indicates a nonspecific band.

To assess the effect of celastrol on NLRP3 inflammasome activation, we first examined whether celastrol could inhibit caspase-1 cleavage and IL-1β secretion. We indeed found that pretreatment of LPS-primed murine peritoneal macrophages with celastrol dramatically reduced IL-1β secretion in a dose-dependent manner (Figure 1A). However, celastrol did not affect the secretion of TNF-α and IL-6, which were inflammasome-independent cytokines (Figure 1B and 1C), suggesting that celastrol inhibited secretion of IL-1β via disrupting inflammasome activation, but did not affect the priming stage under these conditions. Consistently, celastrol dose-dependently suppressed caspase-1 activation and IL-1β maturation, but no effects on pro-IL-1β and pro-caspase-1 expression (Figure 1D). In addition, no obvious cytotoxicity in macrophage was observed after celastrol treatment, indicating that the inhibitory effect of celastrol on IL-1β was not due to the decrease of cell viability (Supplementary Figure 1A). To determinate the effect of celastrol on NLRP3 inflammasome activation was caspase-1 dependent, LPS-primed murine peritoneal macrophages from wide-type and *Caspase-1*^™/™^ mice were treated with celastrol and stimulated with ATP. We found that the effects of celastrol on NLRP3 inflammasome were significantly compromised in *Caspase-1*^™/™^ mice, providing that the effect of celastrol on NLRP3 inflammasome activation depends on Caspase-1 (Supplementary Figure 1B-1D).

Although the above data implied that celastrol did not affect TNF-α when macrophages were treated with celastrol after LPS, previous report has shown that celastrol inhibited NF-κB signaling pathway and the release of TNF-α [29]. We then explored whether celastrol interrupted the LPS-induced priming signal and the activation of inflammasome. As shown in Supplementary Figure 1E, celastrol inhibited TNF-α secretion when macrophages were treated with celastrol before LPS stimulation, although the concentration of celastrol was higher than that used for inflammasome inhibition. In contrast, celastrol had no significant effect on the secretion of TNF-α when macrophages were treated with celastrol after LPS stimulation. Interestingly, we observed that celastrol inhibited caspase-1 cleavage and IL-1β secretion, suggesting that celastrol effectively inhibited both LPS-induced priming and inflammasome activation under different conditions (Supplementary Figure 1F and 1G). In order to address the molecular mechanism underlying the suppression of NLRP3 inflammasome activation by celastrol, cells were treated with celastrol after LPS-stimulated in the later experiments. To further confirm that celastrol could inhibit caspase-1 activation, cell lysates from celastrol-treated LPS-primed macrophages was reacted with recombinant active caspase-1. Consistently, celastrol also markedly decreased the IL-1β cleavage (Figure 1E). Taken together, these results indicate that celastrol exhibits significant inhibitory effects against caspase-1 activation and IL-1β secretion.

### Celastrol inhibits NLRP3 inflammasome activation in various stimulus and different cell types

**Figure 2:**
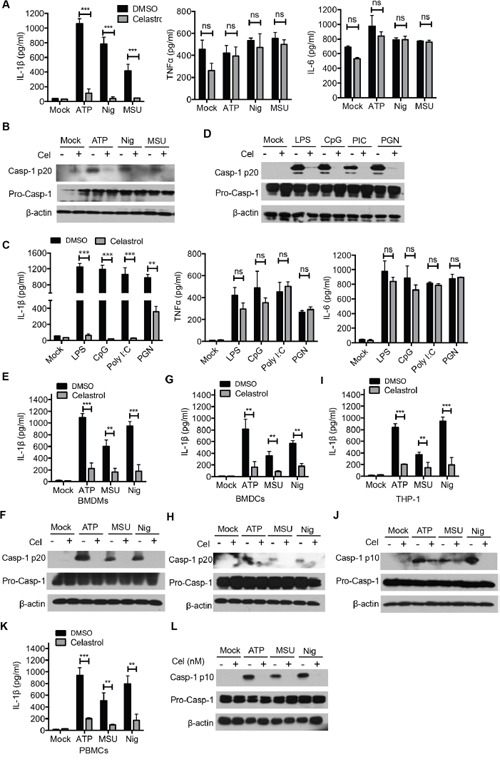
Celastrol inhibits NLRP3 inflammasome activation in various stimulus and different cell types **(A-B)** LPS-primed peritoneal macrophages were treated with the indicated doses of celastrol for 30 min, following by treatment with ATP (30 min), MSU (4.5 h), Nigericin (45 min). Supernatants were analyzed by ELISA for IL-1β, TNF-α and IL-6 release (A). Supernatants and cell extracts were analyzed by immunoblotting (B). **(C-D)** Different TLR ligands-primed peritoneal macrophages (LPS, CpG, Ploy I:C, PGN) were treated with the indicated doses of celastrol for 30 min, following by treatment with ATP for 30 min. Supernatants were analyzed by ELISA for IL-1β, TNF-α and IL-6 release (C). Supernatants and cell extracts were analyzed by immunoblotting (D). **(E-F)** LPS-primed BMDMs were treated with the indicated doses of celastrol for 30 min, following by treatment with ATP, MSU and Nigericin. Supernatants were analyzed by ELISA for IL-1β secretion (E). Supernatants and cell extracts were analyzed by immunoblotting (F). **(G-H)** LPS-primed BMDCs were treated with the indicated doses of celastrol for 30 min, following by treatment with ATP, MSU and Nigericin. Supernatants were analyzed by ELISA for IL-1β secretion (G). Supernatants and cell extracts were analyzed by immunoblotting (H). **(I-J)** PMA-differented THP-1 cells were were treated with the indicated doses of celastrol for 30 min, following by treatment with ATP, MSU and Nigericin. Supernatants were analyzed by ELISA for human IL-1β secretion (I). Supernatants and cell extracts were analyzed by immunoblotting (J). **(K-L)** LPS-primed human PBMCs were treated with the indicated doses of celastrol for 30 min, following by treatment with ATP, MSU and Nigericin. Supernatants were analyzed by ELISA for human IL-1β secretion (K). Supernatants and cell extracts were analyzed by immunoblotting (L).

Aside from ATP, NLRP3 inflammasome can be activated by a wide range of stimuli, such as Nigericin and monosodium urate crystals (MSU) [16, 30]. We performed these NLRP3 agonists and found that celastrol also inhibited IL-1β secretion and caspase-1 activation, but did not affect the secretion of TNF-α or IL-6 and the expression of pro-IL-1β and pro-caspase-1, suggesting that celastrol is a potent and broad inhibitor of NLRP3 inflammasome (Figure 2A and 2B). In addition to LPS, the expression of NLRP3 can be induced by other Toll-like receptor ligands, such as CpG, Poly I:C, PGN. Similarly, the caspase-1 cleavage and IL-1β secretion were also suppressed by celastrol after stimulation with different TLR (Figure 2C and 2D).

As celastrol has multiple effects on inflammation, then we sought to determine whether celastrol inhibit NLRP3 inflammasome activation in other immune cells, apart from murine peritoneal macrophages. LPS-primed mouse bone-marrow-derived macrophages (BMDMs) and bone-marrow-derived dendritic cells (BMDCs) were triggered by ATP, Nigericin and MSU in the presence of celastrol. We found that pretreatment of celastrol effectively inhibited IL-1β secretion and caspase-1 activation in BMDMs (Figure 2E and 2F) and BMDCs (Figure 2G and 2H). Similar results were obtained in the PMA-differentiated human THP-1 cells and human peripheral blood mononuclear cells (PBMCs) (Figures 2I-2L). Taken together, these data indicate that celastrol inhibits NLRP3 inflammasome-mediated IL-1β secretion and caspase-1 activation in both mice and human myeloid cells.

### Celastrol attenuates ROS generation

**Figure 3:**
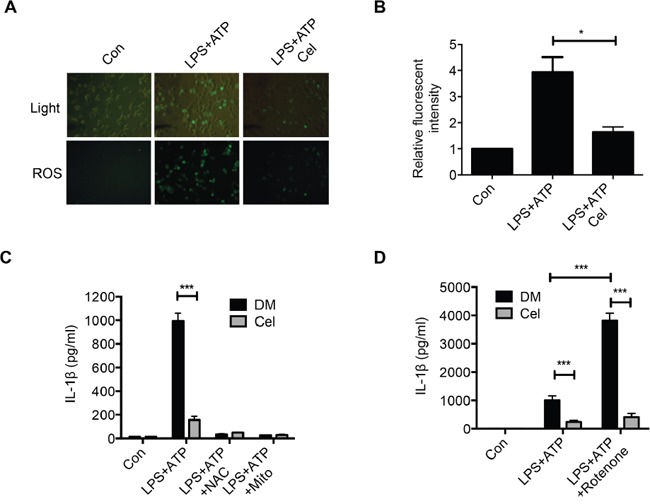
Celastrol attenuates ROS generation **(A-B)** LPS-primed macrophages were stimulated with ATP in the presence or absence of celastrol (DM: DMSO). Cells were then incubated with DCF-DA probe for 1 h for 20 minutes. Fluorescence images were used to exhibit the ROS formation. **(C)** LPS-primed macrophages were treated with NAC or Mito-TEMPO for 30 min before incubation with ATP for 1 h in the presence or absence of celasrol. Supernatants were analyzed by ELISA for human IL-1β secretion. **(D)** LPS-primed macrophages were incubated with rotenone for 30 min before stimulation with ATP for 30 min in the presence or absence of celastrol. IL-1β secretion was analyzed by ELISA.

Intracellular reactive oxygen species (ROS) and mitochondrial DNA (mtDNA) induce the activation of NLRP3 inflammasome signaling via promoting caspase-1 activation and pro-inflammatory cytokines secretion [31]. Indeed, we observed that pretreatment of celastrol impaired the ROS production induced by LPS plus ATP (Figure 3A and 3B). We further used ROS scavengers N-acetyl cysteine (NAC) or mitochondrial ROS scavengers Mito-TEMPO to examine whether the inhibitory effect of celastrol on ROS generation was associated with NLRP3 inflammasome inhibition. Interestingly, both NAC and Mito-TEMPO significantly inhibited IL-1β secretion, implying that celastrol inhibited NLRP3 inflammasome partially through decreasing ROS production (Figure 3C and Supplementary Figure 2A). Rotenone, a mitochondrial membrane depolarization inducer, enhanced IL-1β secretion during LPS plus ATP stimulation [32–33]. We found that celastrol significantly inhibited rotenone-induced overproduction of IL-1β secretion (Figure 3D). These results suggest that celastrol inhibits NLRP3 inflammasome activation in part by suppression of ROS production.

### Celastrol-driven autophagy mediates NLRP3 inflammasome inhibition

**Figure 4:**
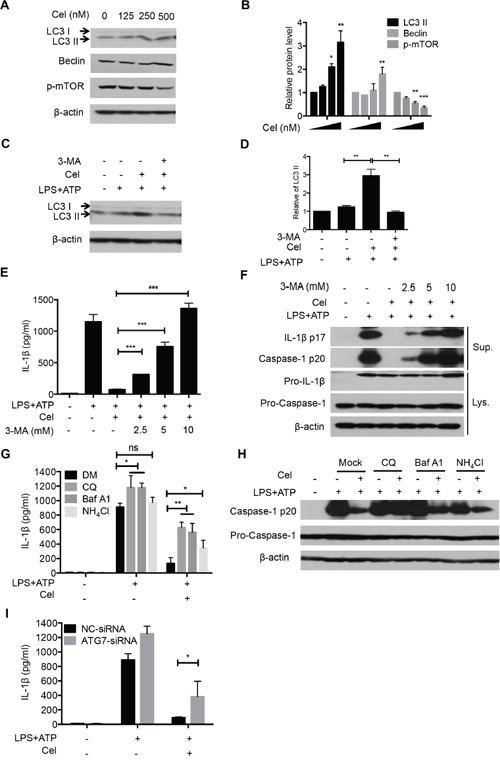
Celastrol-induced autophagy mediates NLRP3 inflammasome inhibition **(A-B)** LPS-primed macrophages were treated with the indicated doses celastrol for 1 h, and cellular levels of LC3-I and LC3-II were determined by immunoblotting. Densitometric analysis was performed to determine the relative ratios of each protein. **(C-D)** LPS-primed macrophages were stimulated with ATP in the presence or absence of celastrol, and cellular levels of LC3-I and LC3-II were determined by immunoblotting. Densitometric analysis was performed to determine the relative ratios of each protein. **(E-F)** LPS-primed macrophages were treated with different doses of 3-MA for 30 min before celastrol treatment, and then stimulated with ATP. Supernatants were analyzed by ELISA for IL-1β secretion (E). Supernatants (SN) and cell extracts (Input) were analyzed by immunoblotting (F). **(G-H)** LPS-primed macrophages were pre-incubated with the autophagy inhibitors Chloroquine, Baf A1 and NH_4_Cl for 1 h, followed by celastrol treatment and then stimulated with ATP. IL-1β production and caspase-1 activation were measured by ELISA and immunoblot, respectively. **(I)** Macrophages were transfected with control siRNA or siRNA targeting ATG7. After 48 h, cells primed with LPS and stimulated with ATP in the presence or absence of celastrol. Supernatants were analyzed by ELISA for IL-1β secretion.

It has been previously reported that enhanced autophagy can inhibit NLRP3 inflammasome activation [34]. Previous studies show that celastrol induces autophagy, which could protect cell injury [13, 35]. We thus sought to determine whether celastrol could inhibit NLRP3 inflammasome activation by promoting autophagy. As expected, celastrol dramatically enhanced the expression of LC3 II in a dose-dependent manner (Figure 4A and 4B). Moreover, celastrol-induced autophagy could be rescued by inhibition of autophagy with 3-Methyladenine (3-MA) (Figure 4C and 4D). Importantly, we observed that pretreatment of 3-MA enhanced IL-1β production and reversed celastrol-induced NLRP3 inflammasome inhibition (Supplementary Figure 2B, Figure 4E and 4F). Similar results were observed when cells were pretreated with other autophagy inhibitors, CQ, Baf A1, NH_4_Cl (Figure 4G and 4H). Consistently, Knockdown of the autophagy gene *Atg7*, which could attenuate autophagy-induction by celastrol, also partially reversed the IL-1β secretion inhibition by celastrol (Figure 4I). These results demonstrate that enhanced autophagy by celastrol contributes to celastrol-mediated NLRP3 inflammasome inhibition.

### Celastrol blocks ASC oligomerization and NLRP3 complex formation

**Figure 5:**
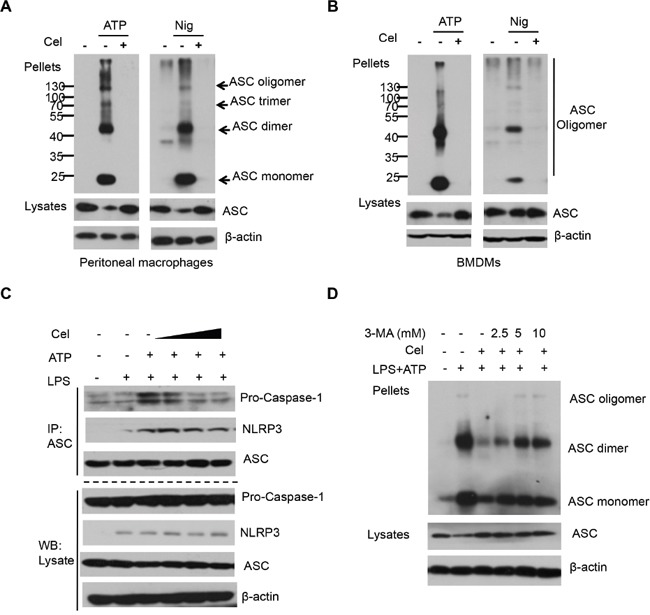
Celastrol blocks ASC oligomerization and NLRP3 complex formation **(A)** LPS-primed peritoneal macrophages were treated with celastrol for 30 min, then stimulated with ATP or nigericin. Immunoblot analysis of ASC in crosslinked pellets and in cell lysates. **(B)** LPS-primed BMDMs were treated with celastrol for 30 min, then stimulated with ATP or nigericin. Immunoblot analysis of ASC in crosslinked pellets and in cell lysates. **(C)** LPS-primed peritoneal macrophages were pretreated with various doses celastrol for 30 min and then stimulated with ATP. The NLRP3-ASC interaction was analyzed by immunoprecipitation and and immunoblotting. **(D)** LPS-primed peritoneal macrophages were pretreated with autophagy inhibitor 3-MA for 30 min, then treated with 250 nM celastrol and then stimulated with ATP. Immunoblotting analysis of ASC in crosslinked pellets and in cell lysates.

ASC pyroptosome are thought to recruit and activate caspase-1, which cleaves the IL-1β and IL-18 [36]. The recent identification of ASC oligomerization is a key event in NLRP3 inflammasome activation [17, 27]. To investigate whether inhibition of ASC oligomerization is involved in celastrol-mediated NLRP3 inflammasome activation, we performed ASC pyroptosome isolation experiment, and found that ASC condensed into dimers, trimmers and oligomers after stimulation with ATP or Nigericin in LPS-primed macrophages. Interestingly, the ASC oligomerizations were significantly disrupted by celastrol (Figure 5A and 5B). We next examined whether celastrol could directly interrupt NLRP3-ASC interaction. As shown in Figure 5C, the interaction of NLRP3-ASC and ASC-caspase-1 were obviously attenuated by celastrol. We further investigated whether celastrol-induced autophagy affect ASC oligomerizations. The results showed that 3-MA significantly blocked the inhibitory effect on ASC oligomerization by celastrol (Figure 5D and Supplementary Figure 2C). These data suggest that celastrol inhibits NLRP3 inflammasome activation through disrupting NLRP3-mediated ASC complex formation.

### Celastrol ameliorates LPS-induced septic shock

**Figure 6:**
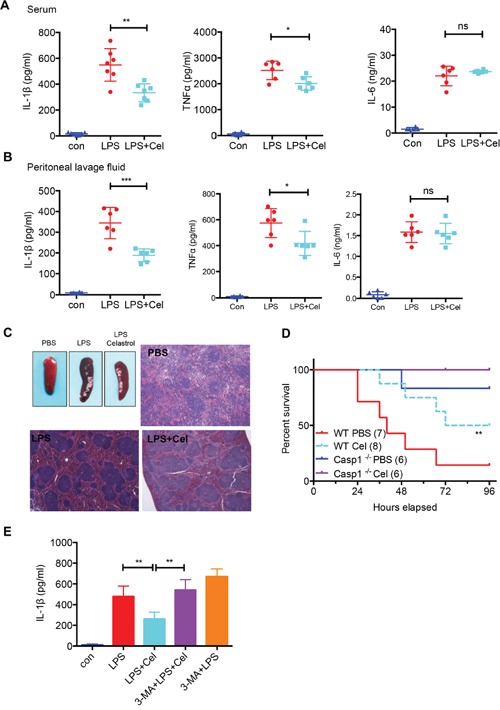
Celastrol ameliorates LPS-induced septic shock **(A)** Serum levels of IL-1β, TNF-α and IL-6 from C57BL/6 mice pretreated with celastrol (1 mg/kg of body weight) or vehicle control as measured by ELISA 4 h after i.p. LPS injection (30 mg/kg of body weight) (n=6). **(B)** Production of IL-1β in peritoneal lavage fluid at 6 h after intraperitoneal injection of LPS (30 mg/kg of body weight) without or with celastrol (1 mg/kg of body weight). **(C)** Representative spleen appearance and histology of control, LPS-treated and LPS plus celastrol-treated mice. **(D)** Survival of mice (wild-type and *Casp1* ^™/™^ mice) intraperitoneally injected with LPS (45 mg/kg body weight) with or without celastrol (1 mg/kg of body weight) (n=6-8). **(E)** Mice pre-treated by daily intraperitoneally injection of 3-MA (15 mg/kg) for 2 days prior to LPS (30 mg/kg of body weight) and celastrol (1 mg/kg of body weight) treatment. After 4 h, the surum were collected and detected by ELISA.

The secretion of IL-1β induced by intraperitoneal injection of LPS is associated with the NLRP3 inflammasome activation [17, 37]. We then treated mice with LPS to induce septic shock mouse model, and evaluated the effect of celastrol in this model. Interestingly, we found that celastrol significantly reduced the level of IL-1β and mildly inhibited TNF-α in serum, whereas no effect on IL-6 was observed (Figure 6A). Similar results were found in the peritoneal lavage fluid (Figure 6B). Histological examination revealed that administration of celastrol attenuated LPS-induced splenomegaly and congestion (Figure 6C). To further determine that celastrol protects mice from LPS-induced septic shock, we assessed survival rate in wild-type or *Caspase-1*^™/™^ mice after LPS treatment in the presence or absence of celastrol. As expected, LPS-treated wild type mice has decreased survival, compared with celastrol–treated mice or *Caspase-1*^™/™^ mice (Figure 6D), implying that celastrol can attenuate NLRP3 inflammasome activation in this model.

To address the requirement of autophagy for anti-systemic inflammation effect of celastrol, mice were injected with 3-MA before celastrol and LPS treatment, then we analyzed IL-1β in serum. Intriguingly, 3-MA treatment could impair celastrol-induced NLRP3 inflammasome inhibition (Figure 6E), indicating that autophagy plays a critical role in celastrol-induced NLRP3 inflammasome inhibition in systemic inflammation.

### Celastrol prevents DSS-induced colitis through inhibition of NLRP3 inflammasome activation

**Figure 7:**
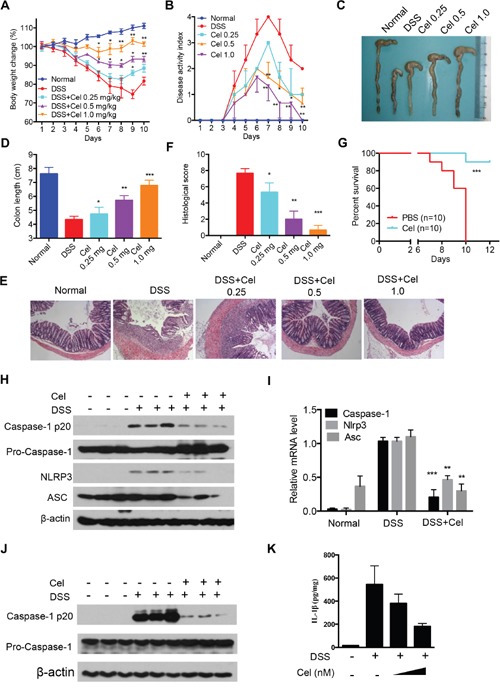
Celastrol ameliorates DSS-induced colitis in mice **(A-D)** Mice were given 3% DSS in their drinking water for 7 days and then provided with water for another 2 days before being sacrificed. Celastrol (0.25, 0.5, 1.0 mg/kg) were given daily. (A) Changes in the body weights of mice were measured (A), disease activity index (DAI) was calculated (B), and the colon lengths of the mice were measured (C-D) during the experiment (n=6-8). **(E-F)** Serial sections of paraffin-embedded colon tissues were stained with H&E. **(G)** C57BL/6 mice were fed a 5% DSS solution in drinking water with celastrol (1.0 mg/kg) or vehicle for 7 days. Survival was monitored until day 12 after the start of DSS. **(H)** Protein levels of cleaved caspase-1, pro-caspase-1, NLRP3 and ASC were determined by immunoblotting from colon tissues. **(I)** RNA was extracted from colonic tissues, and mRNA expressions were determined. **(J)** Mice were given 3% DSS in their drinking water for 7 days. Celastrol (1.0 mg/kg) were given daily. Macrophages from mice peritoneal lavage fluid were stimulated with ATP (5 mM, 30 min). Proteins were analyzed by immunoblotting. **(K)** Peritoneal macrophages isolated from C57BL/6 mice were treated with celastrol (50 and 100 nM) in the presence of 10 mg/ml DSS for 24 h. Production of IL-1β by peritoneal macrophages was determined by ELISA.

Human inflammatory bowel disease (IBD) is a common chronic and recurrent inflammation, and increasing evidence supports that the NLRP3 inflammasome is implicated in the development of IBD [38]. In the acute dextran sodium sulfate (DSS) model, mice develop acute colitis, such as body weight loss, shortened colon length, diarrhea and intestinal bleeding [39]. We herein investigated whether celastrol can suppress DSS-induced colitis via inhibition of NLRP3 inflammasome activation. Interestingly, colitis was ameliorated by celastrol, suggesting that celastrol has a beneficial effect on DSS-induced colitis (Figure 7A-7D). The histological results confirmed that a significant reduction of colonic damage by celastrol treatment (Figure 7E and 7F). More importantly, celastrol significantly increased the survival rate after DSS administration compared to control group (Figure 7G). Furthermore, we found that the mRNA expression of IL-1β, TNF-α, IL-6, IL-17A, IFNg, COX-2, iNOS, CCL2 and CCL5 in colons were significantly inhibited by celastrol treatment (Supplementary Figure 3A). ELISA results also showed that the levels of IL-1β, TNF-α and IL-6 were markedly suppressed (Supplementary Figure 3B). Moreover, protein expression of iNOS, COX-2 and p-STAT3 in colonic tissue were remarkably decreased after celastrol treatment (Supplementary Figure 3C). These results indicate that celastrol significantly prevents DSS-induced colitis.

We next explore whether celastrol inhibit DSS-induced colitis via inhibition of NLRP3 inflammasome activation. The increasing levels of cleaved of caspase-1, NLRP3 and ASC induced by DSS were significantly impaired by celastrol treatment (Figure 7H and Supplementary Figure 3D). Moreover, celastrol treatment markedly decreased mRNA expression caspase-1, NLRP3 and ASC in colon tissue (Figure 7I). In addition, celastrol treatment dramatically decreased caspase-1 cleavage in macrophages from DSS-fed mice (Figure 7J). *In vitro*, celastrol also suppressed the IL-1β production in macrophages (Figure 7K). These data suggest that celastrol ameliorates DSS-induced colitis through inhibiting of NLRP3 inflammasome activation. Previous studies have shown that autophagy is associated with DSS-induced colitis [40–41]. Given that the induction of autophagy might play protective role after celastrol treatment, we found that chloroquine (CQ) partly impaired celastrol-mediated protective effects in DSS models (Supplementary Figure 4A-4D). Interestingly, celastrol-induced inhibition of IL-1β secretion and NLRP3 inflammasome activity were impaired by CQ treatment *in vivo* (Supplementary Figure 4E-4G). Taken together, these results suggest that celastrol-driven autophagy-mediated inhibition of NLRP3 inflammasome is responsible for the protection of mice against DSS-induced colitis.

## DISCUSSION

Celastrol exhibits potential antitumor effect along with proteasome inhibition and HSP90 inhibition [1, 42]. In recent years, the beneficial effect of celastrol has emerged in a variety of diseases, suggesting that celastrol is a potential drug for preventing inflammatory and disorder diseases. The understanding of potential anti-inflammatory mechanisms is beneficial for clinical application of celastrol. In the present study, we show that a new anti-inflammation mechanism for celastrol carrying reducing IL-1β secretion via autophagy-mediated NLRP3 inflammasome inhibition.

Increasing evidence implying that many regulatory mechanisms have been involved in NLRP3 inflammasome activation, such as elevated ROS levels, mitochondria damage and autophagy [43]. ROS is involved in the priming and activation of NLRP3, leading in caspase-1 activation and IL-1β secretion [44]. Our results suggested that celastrol decreased ROS generation and suppressed IL-1β secretion. Recently published reports have found that celastrol targets multiple thiol redox-related proteins, thus leading to decreased ROS production [7, 9]. These results indicate that the effect of celastrol on NLRP3 inflammasome inhibition is likely to be mediated by suppressing ROS generation indirectly. Autophagy as a cellular quality control system facilitates the turnover of damaged proteins and organelles during inflammation and immune responses [45]. Accumulating evidence suggests that autophagic dysfunction is associated with aging and human diseases, including cancer, and neurodegenerative disorders [46]. Deletion of autophagy-associated genes is associated with caspase-1 activity and IL-1β secretion [34]. Notably, celastrol activates caspases and induces cell death at high concentrations [4]. However, at the nanomolar concentrations, it exhibits anti-oxidant and neuro-protective capabilities [13, 47]. Our results are consistent with those of reports demonstrating that autophagy signaling might contribute to celastrol-mediated anti-inflammatory properties. Moreover, Autophagy can clear dysfunctional or misfolded protein aggregates that caused by overproduction of ROS, while autophagy activation could prevent ROS production [48], which indicates autophagy activation impairs ROS generation during inactivation of NLRP3 inflammasome induced by celastrol. The details need to be further investigated in the future.

The LPS-induced endotoxic shock is associated with NLRP3 inflammasome activation, as has been universally used by researchers [17, 26, 49]. NLRP3 deletion ameliorated the LPS-induced endotoxic shock [50]. Our results showed that celastrol treatment significantly relieved IL-1β secretion and improved the survival of mice challenged by LPS. Increasing evidence imply that activation of NLRP3 inflammasome is involved in DSS-induced colitis, which characterized by weight loss, bloody faeces and inflammation, although the precise mechanisms remains controversial [18, 39]. Studies have reported that *Nlrp3*^™/™^ mice are more susceptible to colitis and colon tumorgenesis [39, 51]. On the other hand, Bauer et al. reported that *Nlrp3*^™/™^ mice developed a less severe colitis [18]. The controversial role of NLRP3 in gene-deleted mouse models may be explained by differences in the colony microflora between particular animal facilities. Herein, we found that celastrol significantly ameliorated colitis carrying the suppression of cleaved caspase-1 and IL-1β secretion. These data suggest that celastrol might protect inflammation by inhibiting NLRP3 inflammasome activation *in vivo*.

In summary, our findings demonstrate that celastrol inhibits NLRP3 inflammasome activation through autophagy activation. The data also suggest that celastrol is benefit for the treatment of LPS-induced systemic inflammation and DSS-induced colitis. Considering the function of NLRP3 inflammasome in inflammatory diseases, celastrol might have potential clinical application in NLRP3 inflammasome-driven inflammatory diseases.

## MATERIALS AND METHODS

### Reagents

LPS, ATP, Nigericin, uric acid, NAC, PolyI:C, and PMA were purchased from Sigma. Dextran Sulfate Sodium Salt (DSS) was from MP Biomedicals. The following antibodies were used for western blotting: IL-1β (Santa Cruz), ASC (Santa Cruz), human caspase-1 (Santa Cruz), iNOS (Santa Cruz), NLRP3 (R&D), mouse Caspase-1 (Adipogen), LC3 (Sigma), β-actin (Sigma), COX-2 (Cell Signaling technology, CST), p-STAT3 (CST). Cell viability was determined by measuring ATP levels using Cell Titer-Glo kit (Promega).

### Mice

*Caspase-1*^™/™^ mice were provided by Dr. Guangxun Meng (Institute Pasteur of Shanghai, Chinese Academy of Sciences). All mice were C57BL/6 background and maintained in specific pathogen-free (SPF) facilities. All animal experiments were approved by the guidelines of the Institutional Animal Care and Use Committee of the Institute for Nutritional Sciences, Shanghai Institutes for Biological Sciences, Chinese Academy of Sciences (CAS).

### Cell preparation and stimulation

Human THP-1 cells were grown in RPMI 1640 medium containing 10% FBS, 1% penicillin/streptomycin (P/S) and 50 μM β-mercaptoethanol. To make macrophages, THP-1 cells were differentiated for 3 hr with 100 nM PMA. Peritoneal macrophages were prepared from C57BL/6 mouse. Briefly, mice were intraperitoneal injected with 4% thioglycollate, and the peritoneal cells were isolated from the peritoneal cavity at day 3 post injection. The cells were plated in RPMI 1640 medium supplemented with 10% FBS and 1% penicillin/streptomycin (P/S) for 6 h. Floating cells were removed and washed three times with PBS. The remaining adherent cells were used as the peritoneal macrophages. The bone marrow-derived macrophages (BMDMs) and bone marrow dendritic cells (BMDCs) were derived from femoral and tibia of C57BL/6 mouse and removed the red blood cells. The bone marrow cells were cultured in RPMI 1640 medium complemented with 10% FBS, 2 mM L-glutamine, 1 mM sodium pyruvate, 1% penicillin/streptomycin (P/S) and 50 ng/ml murine M-CSF. The medium was changed every 2 days. At day 7 *in vitro*, the BMDM cells were collected for experiment treatments. The BMDCs were differentiated from bone marrow cells were RPMI 1640 medium complemented with 10% FBS, 2 mM L-glutamine, 1 mM sodium pyruvate and 50 ng/ml murine GM-CSF.

### Elisa

Supernatants from cell culture, serum and tissue were assayed for mouse IL-1β, TNF-α, IL-6 and human IL-1β (eBioscience) according to manufacturer’s instructions.

### siRNA synthesis and transfection

Cells were plated in 24-well plates and then transfected with 50 nM siRNA using Lipofectamine 3000 Transfection Reagent (Invitrogen).

### ASC pyroptosome detection

Macrophages were plated in 6-well plates overnight. The medium was replaced and cells were primed with 500 ng/ml LPS for 3 h, and then treated with different stimulus. The supernatants were removed and cells were washed twice with ice-cold PBS. Cells were harvested, lysed in 500 μL buffer containing 20 mM HEPES-KOH (PH 7.5), 150 mM KCl, 1% NP-40, 0.1 mM PMSF and a protease inhibitor mixture and sheared ten times through a 21-gauge needle. 50 μL of lysate were collected for western blotting. The cell lysates were centrifuged at 5 000 g for 10 min at 4°C. The pellets were washed twice with ice-cold PBS and resuspended in 500 μL PBS. The resuspended pellets were crosslinked with DSS (4 mM) for 30 min at room temperature with rotation. The samples were centrifuged at 5 000 g for 10 min at 4°C and the crosslinked pellets were resuspended in 30 μL SDS buffer. Samples were boiled 7 min at 98°C and analyzed by immunoblotting.

### Immunoblot analysis and Immunoprecipitation

Cells were lysed in RIPA buffer containing 50 mM Tris-HCl (pH 7.4), 150 mM NaCl, 1% Triton X-100, 1% sodium deoxycholate, 0.1% SDS, 1 mM Na_3_VO_4_, NaF 1 mM, a cocktail of 1 mM PMSF and 1 mM protease inhibitors. The lysates were centrifuged at 12 000 g for 10 min at 4°C. Protein concentrations were measured using a spectrophotometer (Thermo Fisher Scientific, USA). The samples were boiled at 98°C for 7 min, chilled on ice and separated using 10% SDS-PAGE electrophoresis and then transferred to nitrocellulose membrane (Millipore Corporation, USA) at 100 V for 2 h. The membrane was blocked in 5% no fat milk/PBST and incubated with primary antibody overnight at 4°C. β-actin was used as a loading control. The membrane was washed with 0.05% Tween-20/PBS and subsequently incubated with an HRP-conjugated secondary antibody (CST, Beverly, MA) that was detected using a chemiluminescent substrate (Thermo Fisher Scientific, USA). For co-immunoprecipitation assay, cells were stimulated, lysed with lysis buffer on ice for 30 min and then centrifuged at 12 000 g for 10 min at 4°C. The supernatant lysates were collected and incubated with the indicated antibody at 4°C overnight. Protein A/G-agarose beads were added and incubated with another 4 h at 4°C. The beads were washed with lysis buffer 6 times by centrifugation at 1 000 g for 5 min at 4°C and then performed as above.

### Real-time PCR

Total RNA was extracted from cells using Trizol Reagent (Invitrogen). First-strand cDNA was synthesized from 1 μg total RNA using PrimeScript RT Master Mix (Takara) according to the manufacturer’s guidelines. Quantitative PCR was performed with SYBR-Green premix (Takara) and detected by Real Time PCR System (StepOne, Applied Biosystems). LC32 was used as an internal control gene.

### LPS-induced septic shock

Mice were injected intraperitoneally (i.p.) with 1 mg/kg celastrol or vehicle control 1 h before injection of 25 mg/kg LPS. After 4 h, the serum and the peritoneal cavities were collected and the levels of cytokines were measured by ELISA. To induce septic shock, mice were injected intraperitoneally with LPS (50 mg/kg), and the mortality was monitored at regular intervals.

### DSS-induced murine colitis

For induction of colitis, male mice were administered 3% DSS in the drinking water for 7 days, followed by 2 days of normal drinking water. Normal mice were given regular water. Celastrol were given 2 days prior to colitis. Mice were monitored for body weight, bleeding and stool daily. The weight at the beginning of DSS treatment was normalized as 100%. The disease activity index (DAI) was recorded as the mean value of the following parameters: normal stools (0), soft stools (1), soft stools and slight bleeding (2), loose stools and slight bleeding (3), gross bleeding (4). At day 10, mice were sacrificed, the colons were measured and collected for histological analysis, RNA and protein extraction. The histological evaluation of H&E –stained was calculated as follows: no inflammation (0), low leukocyte (1), moderate leukocyte (2), high leukocyte, moderate goblet loss and loss of crypts (3), massive goblet loss and loss of crypts (4).

### Statistical analysis

All data were expressed as the mean ± SEM. Statistical analysis were performed with the T-test for two groups or one-way ANOVA for multiple groups (used by GraphPad Software). Significance was defined as p< 0.05.

## SUPPLEMENTARY MATERIALS FIGURES


