# Supplementary material for: Celastrol ameliorates inflammation through inhibition of NLRP3 inflammasome activation
Source: Oncotarget. 2017 Jun 27;8(40):67300–14. doi: 10.18632/oncotarget.18619 (PMC5620174; doi:10.18632/oncotarget.18619)
Supplement: Supplementary file 1 [file oncotarget-08-67300-s001.pdf]

Celastrol ameliorates inflammation through inhibition of NLRP3 inflammasome activation

SUPPLEMENTARY FIGURES

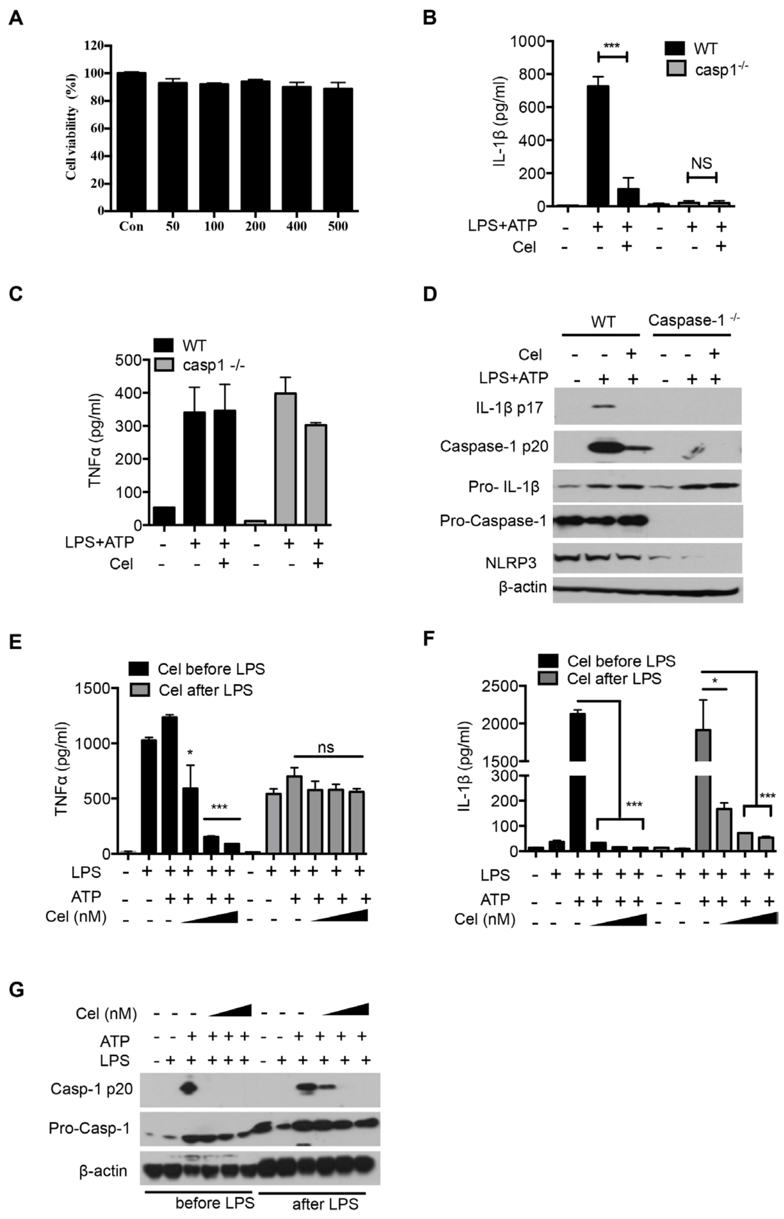

Supplementary Figure 1: Celastrol suppresses NLRP3 inflammasome-mediated caspase-1 activation and IL-1β secretion, Related to Figure 1. (A) LPS-primed peritoneal macrophages were treated with the indicated concentrations of celastrol for 12 h, Cell viability was determined. **(B-C)** ELISA of IL-1β (B) and TNF-α (C) in supernatants from LPS-primed peritoneal macrophages of wild type or casp-1$^{-/-}$ mice treated with celastrol (250 nM) for 30 min and stimulated with ATP. **(D)** Immunoblot analysis of IL-1β (p17) and cleaved caspase-1 (p20) in culture supernatants of LPS-primed peritoneal macrophages treated celastrol (250 nM) for 30 min and stimulated with ATP, and immunoblot analysis of Pro-IL-1β and Pro-caspase-1 in lysates of those cells. **(E-G)** Peritoneal macrophages were treated with different doses of celastrol for 30 min and then stimulated with LPS for 3 h (Cel before LPS), or peritoneal macrophages were primed with LPS for 3 h and then treated with different doses of celastrol for 30 min (Cel after LPS). After that, the cells were stimulated with ATP. Supernatants were analyzed by ELISA for TNF-α (E) and IL-1β (F) release. Cleaved caspase-1 (p20) in supernatants and Pro-caspase-1 in cell extracts were analyzed by immunoblotting (G).

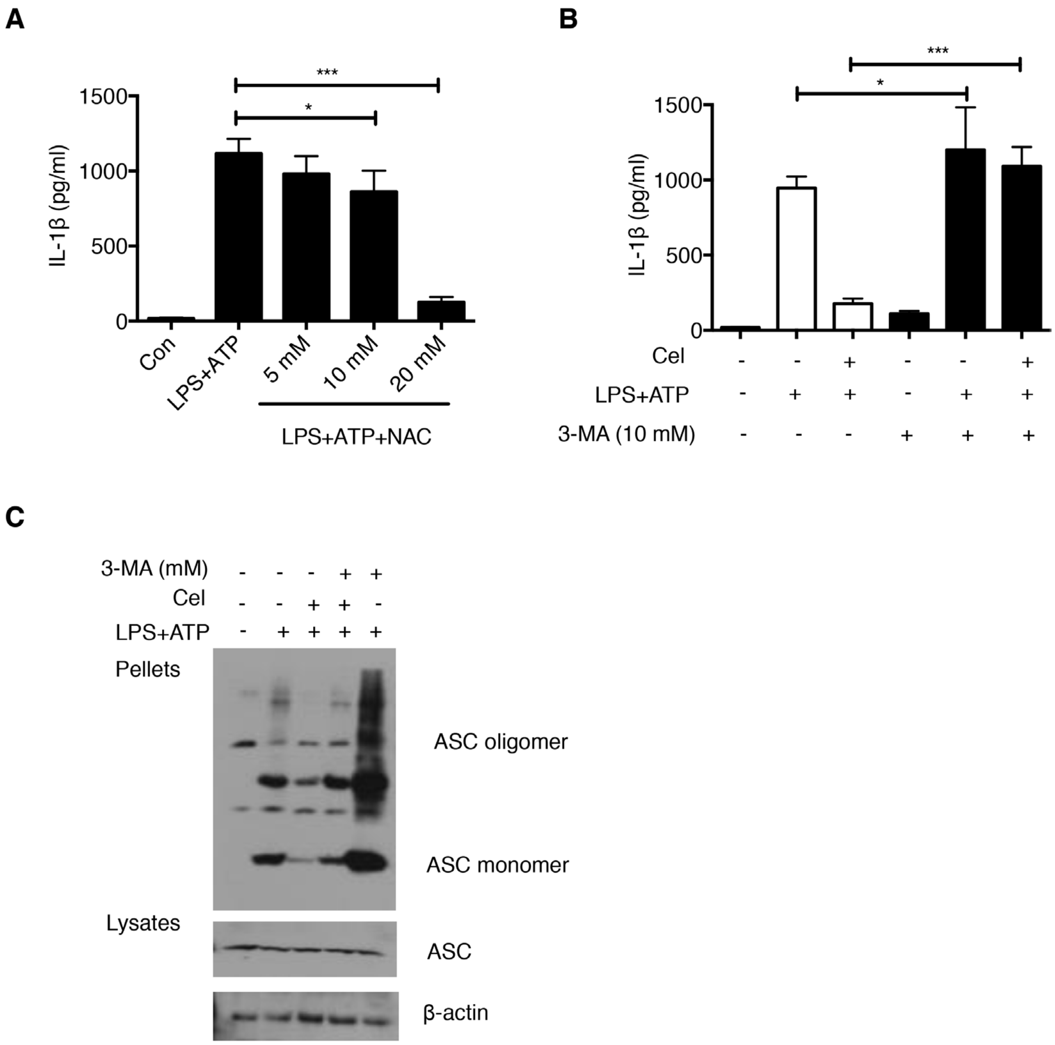

Supplementary Figure 2: Celastrol-mediated NLRP3 inflammasome inhibition are associated with autophagy, Related to Figures 3, 4, 5. (A) LPS-primed peritoneal macrophages were treated with various doses of NAC(5, 10, 20 mM) for 30 min, following by treatment with ATP for 30 min. Supernatants were analyzed by ELISA for IL-1β release. **(B)** ELISA of IL-1β in supernatants from LPS-primed peritoneal macrophages pretreated with 3-MA for 30 min, treated with celastrol for 3 hr and stimulated with ATP. **(C)** LPS-primed peritoneal macrophages were pretreated with 3-MA for 30 min, then treated with 250 nM celastrol and then stimulated with ATP. Immunoblotting analysis of ASC in crosslinked pellets and in cell lysates.

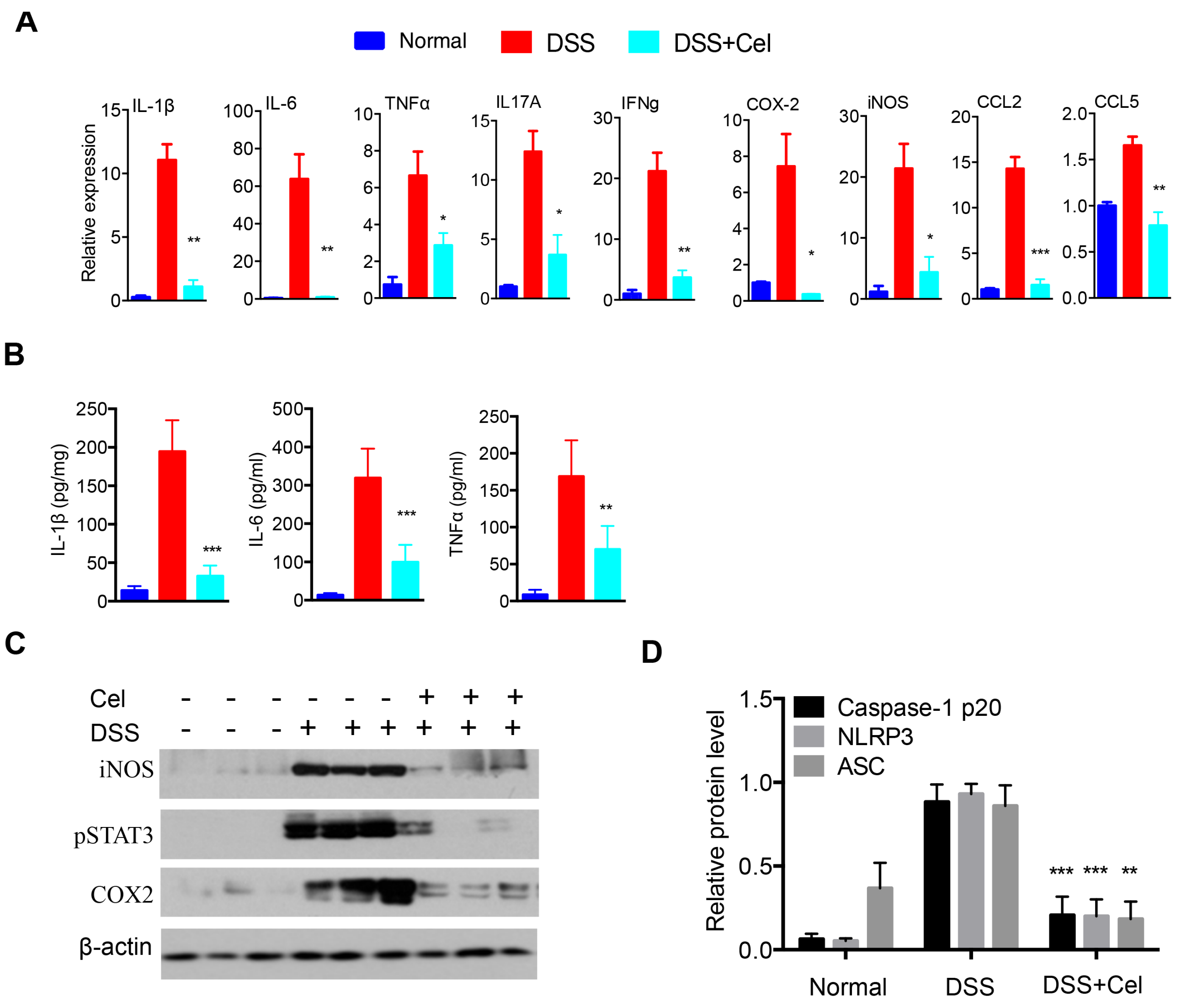

Supplementary Figure 3: Celastrol exhibits protective effect on mice with DSS-induced experimental NLRP3 inflammasome-related colitis, Related to Figure 7. **(A)** The mRNA expression of IL-1β, IL-6, TNF-α, IL-17A, IFNg, COX-2, iNOS, CCL2 and CCL5 in colonic tissues was determined by real-time PCR, and the expression levels were normalized to L32. **(B)** Protein levels of cytokines including IL-1β, IL-6 and TNF-α in colonic homogenate were determined by ELISA. **(C)** The expressions of iNOS, p-STAT3 and COX-2 in colonic tissues were examined by immunoblotting. **(D)** Protein levels of cleaved caspase-1, pro-caspase-1, NLRP3 and ASC were determined by immunoblotting. Densitometric analysis was performed to determine the relative ratios of each protein.

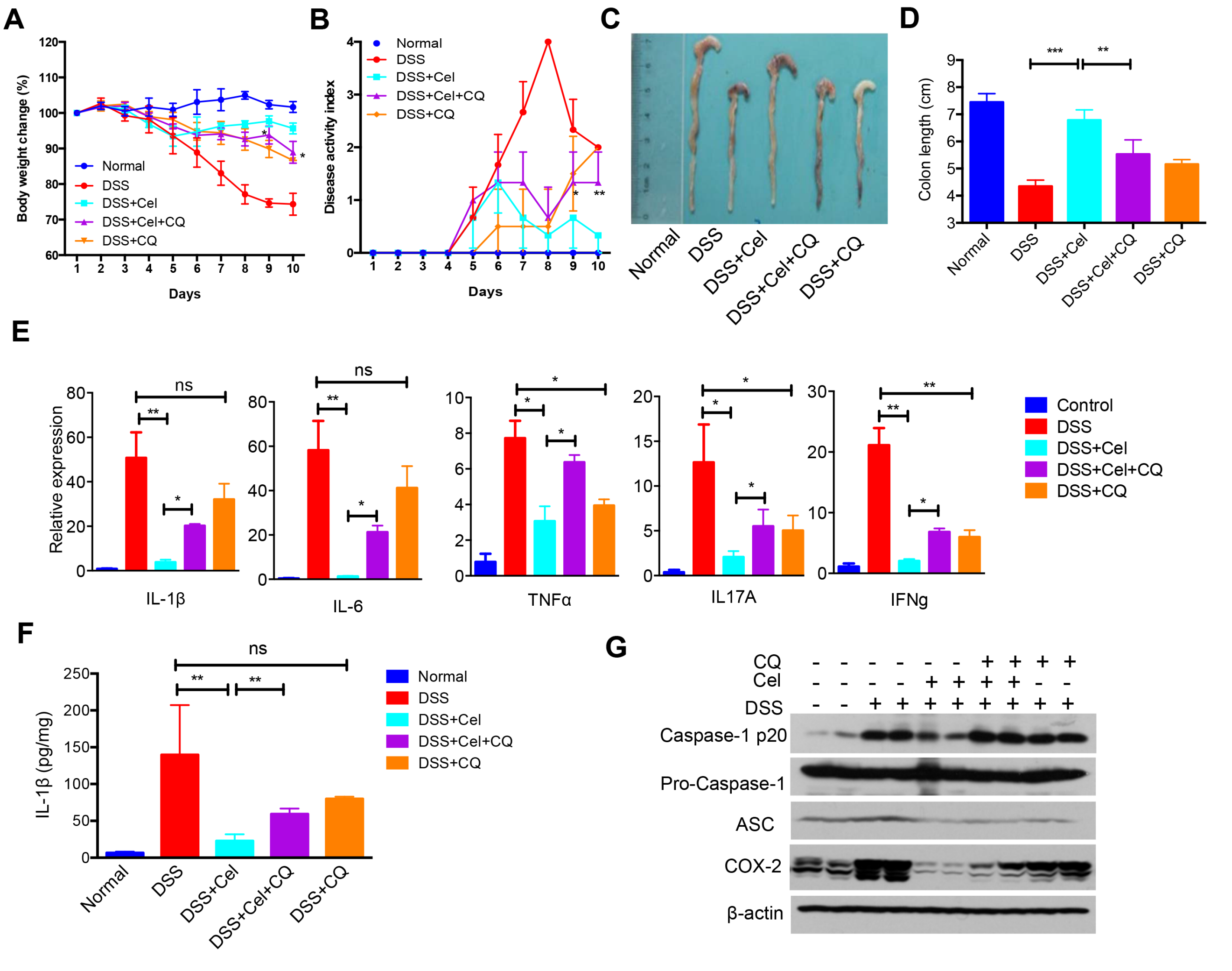

Supplementary Figure 4: Celastrol-driven autophagy-mediated NLRP3 inflammasome inhibition is responsible for amelioration of murine models for colitis, Related to Figure 7. Mice were treated with 3% DSS in their drinking water for 7 days to induce colitis and then provided with normal water for another 3 days before being sacrificed (n=6 per group). Celastrol (1 mg/kg) was given once daily. CQ (50 mg/kg) was administered every 2 days. **(A)** Changes of the body weight was measured. **(B)** Disease activity index (DAI) of each group was calculated. **(C-D)** The length of the colon was measured when the mice were sacrificed. **(E)** The mRNA expression of IL-1β, IL-6, TNF-α, IL-17A, IFNg in colonic tissues was determined by real-time PCR, and the expression levels were normalized to L32. **(F)** Protein levels of IL-1β in colon homogenates from DSS-induced mice were examined by ELISA. **(G)** The expressions of Cleaved caspase-1 (p20), Pro-caspase-1, ASC and COX-2 in colonic tissues were detected by immunoblotting.
